# Repurposing biosimilars, rethinking costs: a framework for sustainable drug pricing for repurposed bevacizumab for intravitreal injections

**DOI:** 10.1007/s10198-025-01891-3

**Published:** 2026-02-07

**Authors:** Evert A. Manders, Vincent van Der Wel, Reinier Schlingemann, Carla E.M. Hollak, Saco J. de Visser

**Affiliations:** 1 Medicine for Society, Platform at Amsterdam University Medical Centre, Amsterdam, The Netherlands; 2 Department of Endocrinology and Metabolism, Amsterdam University Medical Centre, Amsterdam, The Netherlands; 3 RARE-NL, Dutch Collaboration of University Medical Centres Serving as a Hub for the National Centre for Future Affordable Sustainable Therapy Development, Amsterdam, The Netherlands; 4 Amsterdam Centre for Health Economics (ARCHE), Faculty of Economics and Business, University of Amsterdam, Amsterdam UMC, UvA-EB, University of Amsterdam), SEO Amsterdam Economics, Amsterdam, The Netherlands; 5 Orfenix BV, Leiden, The Netherlands; 6 Ocular Angiogenesis Group, Department of Ophthalmology, Amsterdam University Medical Center, Amsterdam, The Netherlands; 7 Department of Ophthalmology, University of Lausanne, Jules Gonin Eye Hospital, Fondation Aisle des Aveugles, Lausanne, Switzerland; 8 Centre for Future Affordable & Sustainable Therapy Development (FAST), The Hague, The Netherlands

**Keywords:** Drug repurposing, Bevacizumab, Neovascular age-related macular degeneration, Retinal vein occlusion, Diabetic macular oedema, Drug pricing, Pricing strategies

## Abstract

Bevacizumab, originally developed by Genentech under the brand name *Avastin*^®^ as an anti-cancer drug, has gained widespread off-label use in ophthalmology due to its similar mechanism of action to other anti-VEGF treatments and its significantly lower cost compared to available on label alternatives for ophthalmological indications. While off-label bevacizumab has been standard in clinical practice for over a decade, recently, a repurposed formulation (brand name: *Lytenava*^®^, Outlook Therapeutics Ltd), developed specifically for vascular retinal conditions, received marketing approval from the European Medicines Agency. This raises questions about what the price for a repurposed formulation should reasonably be, reflecting the efforts to obtain regulatory approval. This paper examines potential cost-based-plus pricing for such a repurposed formulation of bevacizumab using a novel pricing framework across four scenarios. By evaluating the pricing structure through an analysis of critical cost components, including, among others, research and development expenditures, manufacturing costs, and cost-of-capital, the study proposes a price range of €73 to €177 per injection. The explicit breakdown of these cost components provides valuable insights into the economic structure of repurposed biosimilars like bevacizumab, emphasizing how a cost-based-plus pricing model can support more transparent and informed negotiations between pharmaceutical companies and healthcare payers. Ultimately, this approach contributes to the development of pricing strategies that balance affordability for healthcare systems with sustainable returns for manufacturers while fostering the broader development of repurposed treatments. The findings of this paper aim to advance the dialogue on equitable pricing for repurposed therapies.

**Supplementary Information:**

The online version contains supplementary material available at https://doi.org/10.1007/s10198-025-01891-3.

## Introduction

Bevacizumab is a humanized, full-length monoclonal antibody targeting vascular endothelial growth factor (VEGF). Originally developed by Genentech under the brand name Avastin^®^ to treat breast, lung, and gastrointestinal cancers, its off-label use for neovascular age-related macular degeneration (nAMD, or ‘wet’ AMD), as well as other retinal vascular diseases such as retinal vein occlusion and diabetic macular oedema, became widespread—primarily because of its significantly lower cost compared to other anti-VEGF therapies. In 2005, clinical trials demonstrated the efficacy of ranibizumab, another anti-VEGF agent developed by Genentech under the brand name Lucentis^®^, which was specifically designed to treat nAMD [1]. This led to its approval by the U.S. Food and Drug Administration (FDA) in 2006 and by the European Medicines Agency (EMA) in 2007 [2, 3]. Concurrently, ophthalmologists began using intravitreal bevacizumab to treat nAMD, attracted by its similar VEGF-inhibiting mechanism, structural resemblance to ranibizumab, and substantially lower price [4, 5]. Although subsequent studies confirmed the safety and efficacy of bevacizumab for nAMD [6–9], Genentech did not pursue regulatory approval for this specific indication.

Some critics argue that financial motives influenced the decision not to register Avastin^®^ for nAMD [10], particularly given its lower cost (around €36 per injection [11]) compared to Lucentis^®^ (approximately €517 per injection [12]). Both drugs were developed by Genentech, with Avastin^®^ marketed outside the United States by Roche and Lucentis^®^ distributed by Novartis through a licensing agreement, creating no commercial incentive to pursue approval for bevacizumab in ophthalmology. Other anti-VEGF treatments approved for nAMD, such as Bayer’s aflibercept (Eylea^®^) and Novartis’ brolucizumab (Beovu^®^), are priced at over €700 per injection [11, 12], emphasizing the stark cost contrast among available treatment options.

These price differences have created a unique situation: despite general prescribing guidelines advising against off-label use when licensed alternatives exist, bevacizumab has become the preferred treatment for many ophthalmologists due to its affordability. The World Health Organization (WHO) supported this practice by listing bevacizumab as an essential medicine for nAMD [13, 14]. In the Netherlands, bevacizumab was even incorporated into national treatment guidelines as a first-line therapy [11, 15], largely based on the Dutch BRAMD study [8, 11], which directly compared bevacizumab and ranibizumab. This trial showed that bevacizumab was equally effective but substantially more cost-efficient, leading to its formal recommendation.

The widespread off-label use of bevacizumab has sparked ongoing debate and regulatory scrutiny [16]. In 2014, the Italian Competition Authority (AGCM) fined Roche and Novartis over €90 million each, accusing them of market manipulation by artificially distinguishing between Avastin^®^ and Lucentis^®^—one approved for ophthalmology, the other not—to discourage off-label use of the cheaper alternative. The AGCM argued this led to increased healthcare spending. In 2018, the European Court of Justice upheld this view, ruling that the companies’ agreement could be considered a restriction of competition “by object” [17]. Similarly, in 2020, a group of UK clinical commissioning groups won a legal case allowing them to offer bevacizumab as an option for nAMD treatment, despite objections from Roche and Bayer [18, 19]. Also in 2020, the French Competition Authority fined Novartis, Roche, and Genentech for leveraging their collective dominance to suppress off-label use. However, this decision was overturned by the Paris Court of Appeal in 2023 [20].

Even before Avastin^®^’s European patent expired in 2022, several biosimilars had already been approved by the EMA, although none were registered specifically for nAMD [21, 22]. The landscape shifted significantly in May 2024, when Outlook Therapeutics Ltd. received EMA approval for Lytenava^®^ [23, 24], an intravitreal formulation of bevacizumab developed specifically for nAMD, based on data from the NORSE trials [23–31]. This marked the first officially authorized version of bevacizumab for this indication, raising questions about the future of off-label use, particularly given that such prescribing is often discouraged or restricted once a licensed alternative becomes available.

Although Lytenava^®^’s price in the European Union (EU) is still undetermined, its list price in the UK is reported at £470 per single use vial. This value-based price, recommended by the National Institute for Health and Care Excellence [32], is considerably higher than that of the currently used off-label bevacizumab, potentially straining already limited healthcare budgets (Table 1). While a registered formulation offers regulatory and quality advantages, it is essential to ensure this does not compromise its cost-savings relative to the long-used off-label product. In the Netherlands, where bevacizumab is commonly used off-label, Lytenava^®^ has been placed under the “lock procedure” for expensive hospital medicines due to anticipated budget impact [33] This procedure temporarily excludes high-cost drugs from the basic health insurance package while price negotiations and evaluations of clinical and economic value are conducted.

**Table 1:** Overview of list prices for anti-VEGF treatments in ophthalmology (including off-label options)

Product:	Brand name:	Indication area	Price per injection (€):	Source:
Bevacizumab	Avastin^®^	Malignancies	35.53¹	[11]
	Lytenava^®^	Ophthalmology	470 (£)	[32]
Ranibizumab	Lucentis^®^	Ophthalmology	516.84	[12]
Aflibercept	Eylea^®^	Ophthalmology	806.39	[12]
Brolucizumab	Beovu^®^	Ophthalmology	738.86	[12]

¹ Price based on a 0.1 ml injection vial prepared for nAMD

Given these developments (Table 2), this paper explores a range of sustainable pricing for repurposed bevacizumab using a cost-based plus pricing framework. It assesses both minimum and maximum price points and evaluates the broader economic implications of formal registration and market introduction of bevacizumab for ophthalmologic indications in the Netherlands and the EU.

**Table 2:** Timeline of the bevacizumab debate

Year:	Event:	Source:
2004	Bevacizumab (Avastin^®^) approved by FDA for various cancers.	[40]
2006	Clinical trials show efficacy of ranibizumab (Lucentis^®^) for nAMD.	[1]
	Ranibizumab (Lucentis^®^) approved by FDA for nAMD.	[2]
< 2006	Off-label use of bevacizumab for nAMD due to its VEGF-targeting similarity and lower cost.	[5, 6]
2007	Ranibizumab (Lucentis^®^) approved by EMA for nAMD.	[3]
< 2011	Further clinical trials confirm bevacizumab’s safety and efficacy for nAMD.	[7–9]
2012	Aflibercept (Eylea^®^) approved by EMA for nAMD.	[41]
2014	Italian AGCM fines Roche and Novartis €90 million for artificially distinguishing Avastin^®^ and Lucentis^®^.	[17]
2018	European Court of Justice upholds AGCM’s view, citing restriction of competition.	[17]
2018–2024	Bevacizumab biosimilars are approved for the EU market: Mvasi^®^, Aybintio^®^, Equidacent^®^ (withdrawn), Zirabev^®^, Alymsys^®^, Onbevzi^®^, Oyavas^®^, Abevmy^®^, Vegzelma^®^, Avzivi^®^.	[42–51]
2020	UK clinical commissioning groups win court case allowing bevacizumab as treatment option	[18, 19]
	Brolucizumab (Beovu^®^) approved by EMA for nAMD.	[52]
2020–2023	French Competition Authority fines Novartis, Roche, and Genentech; later overturned in 2023.	[20]
2022	Avastin^®^ patent expires in EU.	[21]
2024	Lytenava^®^ receives EMA approval for intravitreal use in nAMD.	[23, 24]
	NICE recommends Lytenava^®^ with list price of £470 per single-use vial.	[32]

## Methods

To assess the feasibility and implications of cost-based plus pricing for repurposed bevacizumab, this study applies a drug pricing framework across multiple scenarios in both Dutch and broader EU contexts. These scenarios account for variations in development costs, development and recoupment timelines, and profit margins, providing a systematic approach to evaluating pricing outcomes under different assumptions.

### Pricing framework

**Fig. 1:**
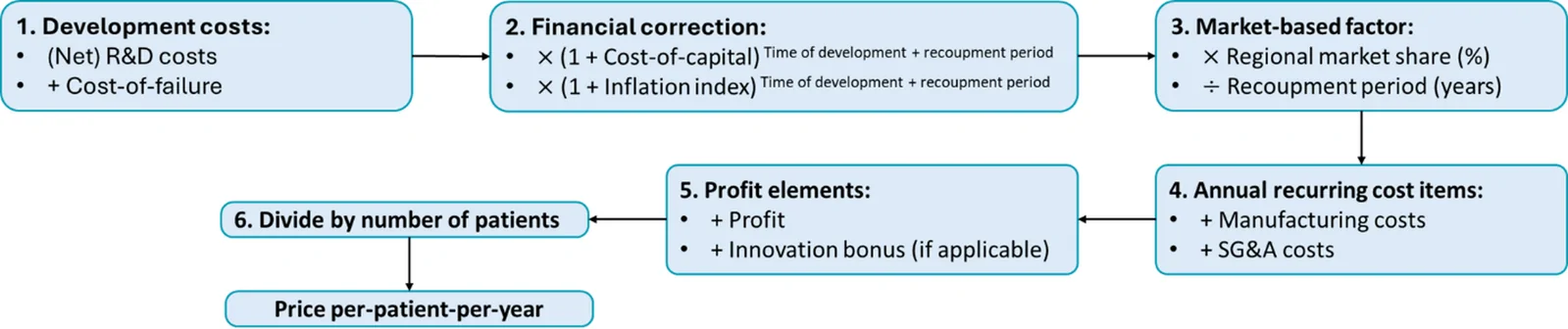
Visual overview of the pricing approach

The pricing structure for repurposed bevacizumab is analysed through a cost-based-plus framework, illustrated in Fig. 1, detailed in Table 3, and defined by the equations below. The framework calculates a price per-patient-per-year (PPPY). Consequently, all components are expressed as the annual amounts to be recovered for a defined target region over a specified recoupment period, providing a transparent and structured basis for assessing treatment costs.

**Table:** 

Equation 1:
Price PPPY = ∑(i = 1)N [(R&D_i_ + CoF_i_ + CoC_i_) × MAF_i_ + MC_i_ + SG&A_i_ + P_i_ + IB_i_]) ÷ ∑(i = 1)N Patients_i_
Where:
Equation 2: R&D_i_ = Lead development costs + Nonclinical costs + In-licensing costs + Clinical costs + Regulatory costs + Legal costs + Product development costs + R&D operational costs + Early and expanded access costs – Public investments & Charitable funding Equation 3*: CoF_i_ = Cost-of-failure correction factor × (R&D_i_) Equation 4: CoC_i_ = Cost-of-capital (development phase) + Cost-of-capital (recoupment phase) Equation 5: Cost-of-capital (development phase) = (R&D_i_ + CoF_i_) × [(1 + WACC_i_)^D_i_ × (1 + GDP_i_)^D_i_ – 1] Equation 6: Cost-of-capital (recoupment phase) = ∑(t = 1)ᴿⁱ [(R&D_i_ + CoF_i_ + CoC_Development) × (1 – t/R_i_) × WACC_i_ × (1 + GDP_i_)^t] Equation 7: MAF_i_ = Regional market share ÷ R_i_Equation 8: MC_i_ = Manufacturing cost per treatment × Manufacturing volume Equation 9: SG&A_i_ = Cost-of-sales + Operational costs + Overhead costs + Post-authorization obligations + Regulatory maintenance + Patent maintenance costs + Licensing obligations Equation 10: P_i_ = Profit margin × [(R&D_i_ + CoF_i_) × MAF_i_ + MC_i_ + SG&A_i_] Equation 11: IB_i_ = Value-driven premium × [(R&D_i_ + CoF_i_) × MAF_i_ + MC_i_ + SG&A_i_]
With:
R&D_i_: (Net) R&D costs for indication i CoF_i_: Cost-of-failure for indication i CoC_i_: Cost-of-capital for indication i MAF_i_: Market allocation factor for indication i MC_i_: Manufacturing costs for indication i SG&A_i_: Selling, general & administrative costs for indication i P_i_: Profit for indication i IB_i_: Innovation bonus for indication i Patients_i_: Number of patients receiving treatment for indication i WACC_i_: Weighted average cost-of-capital for indication i D_i_: Time of development in years for indication i R_i_: Recoupment period in years for indication i GDP_i_: Inflation index (e.g. GDP deflator) for indication i t: Specific year of recoupment (1 ≤ t ≤ R_i_)

* Alternatively, cost-of-failure may be represented as an absolute monetary value instead of using a correction factor

The framework builds on established drug pricing models proposed by the International Association of Mutual Benefit Societies (AIM) [34], Uyl-de Groot and Löwenberg [35], Nuijten and Vis [36], and van der Schans et al. [37], following a detailed analysis of their core components [38], as well as the application of these models in the case of lumasiran [39].

The framework first incorporates the fixed expenditures associated with developing a new molecular entity (NME) or repurposing an established drug. These expenditures include R&D costs as well as the implicit costs of failed projects (cost-of-failure). While R&D expenditures are typically derived from several input parameters (Table 3), in the absence of detailed figures, a lump-sum estimate is assigned for the development of a new formulation of bevacizumab.

**Table 3:** Overview of the frameworks’ components

Category:	Components:	Cost type:	Parameter:	Definition:
1. Development costs	(Net) R&D costs	Fixed	Lead development costs	Costs associated with early discovery, target identification and validation, hit discovery, lead optimization, and candidate selection required to nominate a viable drug candidate for further development.
			Non-clinical development costs	Costs associated with early-stage research activities. Includes discovery, in vitro and in vivo studies, pharmacology, and toxicology assessments.
			In-licensing costs	Costs incurred when acquiring the rights to develop, manufacture, or commercialize a drug candidate from an external entity.
			Clinical development costs	Costs related to conducting clinical trials (Phases I–III), including trial design, site management, investigator fees, patient recruitment, data monitoring, and clinical operations.
			Regulatory development costs	Expenses associated with regulatory strategy, interactions with regulatory authorities, and the preparation of dossiers for marketing authorization, including consultancy services.
			Legal development costs	legal expenses incurred during the development phase to secure product readiness and mitigate launch-related risks. Includes freedom-to-operate analyses, initial patent filing and strategy, legal support for regulatory submissions, compliance reviews, and partnership/contractual negotiations.
			Product development costs	Fixed costs required to convert a drug candidate into a trial-ready and manufacturable product. Includes GMP process development (e.g., synthesis, cell line/vector creation, gene editing protocols), formulation and delivery optimization, analytical method development/validation, production of engineering and clinical trial batches, process validation, equipment qualification, and preparation of CMC documentation (e.g., ASMF, IMPD, BHR, or equivalent dossiers for biologics and ATMPs)
			R&D operational costs	Ongoing expenses that enable the day-to-day functioning of R&D but cannot be directly linked to a specific study or trial. Covers staff salaries, laboratory facilities and utilities, general consumables, project management, and IT systems that support multiple research activities.
			Early and expanded access costs	Costs associated with providing investigational drugs to patients outside of clinical trials before marketing authorization, under expanded access, compassionate use, or early access programs.
		Fixed deduction (cost offset)*	Public investments & charitable funding	External funding from governments, public institutions, or nonprofit organizations that reduces the net private cost of R&D. As these contributions do not always require repayment or return, they may be deducted when calculating recoverable investment.
	Cost-of-failure	Fixed	Cost-of-failure correction factor**	A factor representing the ratio of cost-of-failure to R&D costs, used to account for expenses incurred during the R&D process for drugs that ultimately do not reach the market due to failure at any stage. By distributing these costs across successful products, this factor reflects the inherent financial risk of pharmaceutical innovation.
2. Financial correction factors	Cost-of-capital	Fixed	WACC (%)	A metric representing the average cost a company incurs to finance its operations through a combination of equity and debt. Expressed as a percentage of invested capital, it reflects the blended cost of raising funds from equity investors and creditors. Often used as a discount factor in financial evaluations.
			Cost-of-capital (development phase)	The capital charge applied to out-of-pocket R&D expenditures and cost-of-failure during the development period (from research initiation until market authorization). It reflects the opportunity cost of tying up capital before any revenue is generated.
			Cost-of-capital (recoupment phase)	The capital charge applied during the recoupment period on the outstanding unrecovered balance of R&D investment. It reflects the ongoing financing cost of carrying unrecovered capital until full cost recovery is achieved through sales.
	Inflation index	N/A	GDP deflator (%)	A measure used to adjust nominal GDP to reflect inflation rates, thereby accounting for inflation when setting or evaluating costs.
	Time of development	N/A	Development period (years)	The duration from the initial research phase to obtaining market authorization for a new drug.
3. Market-based factor	Market allocation factor	N/A	Regional market share (%)	The proportion of the revenue base attributed to the target region, used to allocate global development costs. This reflects the region’s relative economic weight and revenue potential.
			Recoupment period (years)	The timeframe within which the company aims to recover its initial investment costs through sales revenue.
4. Annual recurring cost items	Manufacturing costs	Recurring	Manufacturing cost per treatment	The cost associated with manufacturing each individual unit of the product, including raw materials, labour, and overhead expenses directly tied to production.
			Manufacturing volume	The quantity of the product that needs to be produced annually to meet market demand. Influenced by the number of treatments required per-patient-per-year and the number of patients.
	SG&A costs	Recurring	Cost-of-sales	Sales-related expenses, such as distribution, marketing, and sales force efforts.
			Operational costs	Indirect costs associated with ongoing company operations, such as corporate administration, HR, finance, legal, compliance, and IT services.
			Overhead costs	Facility and administrative expenses, including rent, utilities, insurance, and depreciation.
			Post-authorization obligations	Costs incurred after market approval, including participation in patient registries, long-term safety and effectiveness monitoring, and the collection of real-world evidence as required by regulators or payers.
			Regulatory maintenance	Ongoing expenses related to maintaining product authorization, submitting periodic safety updates (PSURs), variations, renewals, and compliance with evolving regulatory requirements.
			Patent maintenance costs	Recurring legal expenses required to preserve IP rights after the initial patent strategy is implemented. Includes renewals, jurisdictional filings, and defence activities such as oppositions.
			Licensing obligations	Incremental legal or financial obligations under licensing agreements, such as royalty payments per unit sold or milestone fees tied to specific development or commercialization events.
5. Profit elements	Profit	Recurring	Profit margin (%)	Bonus added to ensure a baseline level of profitability, supporting financial stability and continued investment.
	Innovation bonus	Recurring	Value-driven premium (%)***	An additional pricing bonus, granted based on the drug’s clinical effectiveness and innovation, reflecting its added therapeutic value and novelty.
6. Patient population	Number of patients	N/A	Number of patients receiving treatment***	The total number of patients expected to receive the drug treatment each year. This figure is shaped by factors such as disease incidence, prevalence, mortality rates, competition, and market penetration.

* While technically income, development cost offsets (e.g., public and charitable contributions) can be accounted for as deductions from private R&D investments

** Alternatively, cost-of-failure may be represented as an absolute monetary value

*** Dynamically adjustable based on emerging (clinical) data

These costs are then adjusted to account for cost-of-capital and inflation, accumulated over both the development phase and the projected recoupment period. Consistent with the AIM model, these adjusted costs are proportionally scaled to reflect the market size of the target region and the anticipated recoupment timeline. This ensures that pricing outcomes capture both the size of the accessible market and the recovery timeframe. For this case, the relative share of either the Dutch or the EU population to the total population of OECD countries (≈ 1.38 billion in 2023 [53]) is used, based on the assumption that OECD countries represent the main market for new drugs due to their high healthcare spending and rapid adoption of new medicines [54].

Subsequently, ongoing annual expenditures are added, including manufacturing costs and selling, general, and administrative (SG&A) expenses. Similar to R&D costs, SG&A expenses are normally composed of multiple parameters (Table 3). However, due to limited transparency, a lump-sum estimate is applied.

A profit margin is then included to provide financial incentives for pharmaceutical companies to invest in drug development. In this framework, the margin is applied to net R&D costs, cost-of-failure, manufacturing costs, and SG&A costs. Development costs are not further capitalized or adjusted for inflation but are scaled according to the regional market share and recoupment period.

Finally, the framework allows for a value-driven innovation bonus to further stimulate and reward therapeutic breakthroughs that deliver substantial clinical benefits compared to existing treatments. This bonus is applied in the same manner as the profit margin. However, in this case, the innovation bonus is omitted, as the ophthalmologic use of bevacizumab is not considered novel.

### Scenario analysis

For repurposed bevacizumab, this analysis applies four scenarios using case-specific assumptions and reference values, assessing both minimum and maximum pricing outcomes for the Dutch context, where off-label use of bevacizumab is already included in clinical guidelines, as well as for the broader EU (Table 4).

**Table 4:** Overview of pricing scenarios for repurposed bevacizumab

Items:	Scenario 1:	Scenario 2:	Scenario 3:	Scenario 4:
1. Development costs				
(Net) research & development costs	€780.900	€780.900	€50 million	€50 million
Cost-of-failure	No risk is assumed, since bevacizumab has been used intravitreally for over a decade	No risk is assumed, since bevacizumab has been used intravitreally for over a decade	3,85 times the assigned (net) R&D costs	3,85 times the assigned (net) R&D costs
**2. Financial correction factors**				
Cost-of-capital	Calculated at a discount rate of 10,5%	Calculated at a discount rate of 10,5%	Calculated at a discount rate of 10,5%	Calculated at a discount rate of 10,5%
Development period (years)	4	4	10	10
**3. Market-based factor**				
Regional market share (%)	4%	100%	4%	32,61%
Recoupment period (years)	10	10	3	3
**4. Annual recurring cost items**				
Manufacturing costs	Benchmarked at €35,53 per tablet and 6 injections per-patient-per-year	Benchmarked at €35,53 per tablet and 6 injections per-patient-per-year	Benchmarked at €35,53 per tablet and 6 injections per-patient-per-year	Benchmarked at €35,53 per tablet and 6 injections per-patient-per-year
SG&A costs	Benchmarked at 90% of manufacturing costs	Benchmarked at 90% of manufacturing costs	Benchmarked at 90% of manufacturing costs	Benchmarked at 90% of manufacturing costs
**5. Profit elements**				
Profit (%)*	8%	8%	25%	25%
Innovation bonus	None, as intravitreal bevacizumab is already well established	None, as intravitreal bevacizumab is already well established	None, as intravitreal bevacizumab is already well established	None, as intravitreal bevacizumab is already well established
**6. Patient population**				
Number of patients	19.019	380.000	19.019	380.000

*Applied to non-capitalized development costs (net R&D costs and cost-of-failure), not adjusted for inflation, but corrected for the regional market share and the assigned recoupment period. This bonus is further granted on top of the out-of-pocket annual recurring cost items. Reflects the annual amount that is provides as bonus each year

The first two scenarios estimate the minimum costs required to repurpose bevacizumab for nAMD and, subsequently, the minimum costs necessary to bring a registered product to market. These scenarios focus primarily on administrative and regulatory compliance expenses. Given that bevacizumab has been used off-label for over a decade [4–6] with well-established efficacy and safety in the treatment of nAMD, it is assumed that formal registration for commercial use would not require additional clinical trials beyond those typically required for biosimilars. For substances with a long history of safe and effective use, the EMA permits marketing authorisation via the well-established use pathway, allowing applicants to rely on published scientific evidence [55]. Therefore, development costs in these minimum scenarios are limited to regulatory and administrative fees. A modest profit margin is assigned with an assumed recoupment period of 10 years during which initial costs would need to be recovered, consistent with evidence that roughly half of new drugs reach breakeven within this timeframe [56].

As repurposing bevacizumab for a new therapeutic indication entails more stringent regulatory requirements than those applied to biosimilars for oncology indications, the latter two scenarios reflect a more rigorous development pathway, explicitly incorporating R&D costs and cost-of-failure. Even though the molecule remains unchanged, demonstrating efficacy and safety in a new clinical context, in this case ophthalmology rather than oncology, requires dedicated clinical trials. As a result, the regulatory burden, and the associated development costs, exceed those incurred for biosimilar approval for existing indications [55]. These maximum scenarios also assume a higher profit margin and a shorter recoupment period of three years.

In the minimum-cost scenarios, a four-year development timeline is assumed. In the maximum-cost scenarios, a ten-year development period is assumed, reflecting the full timeline from the initiation of the first NORSE study to EMA approval of Lytenava^®^ [23–25].

To estimate manufacturing costs, all scenarios benchmark against the current annual price of off-label Avastin^®^ [11], which is broadly comparable to the market prices of approved bevacizumab biosimilars [57]. While this benchmark likely overstates true production costs, it offers a transparent reference point, as biosimilars authorised for oncological indications are typically manufactured using platforms similar to those required for ophthalmic formulations such as Lytenava^®^. However, it is important to note that the market price of bevacizumab biosimilars includes non-negligible regulatory, legal, and product development costs (Table 3). Although biosimilar approval pathways are less stringent than those for original biological products, they still require comprehensive comparability exercises, including extensive analytical characterisation and clinical trials to establish biosimilarity in terms of efficacy, safety, and pharmacokinetics relative to the reference product. Therefore, using biosimilar prices as a proxy for manufacturing costs alone results in a systematic overestimation, since such prices implicitly cover a broader range of expenditures and a profit margin sufficient to sustain commercial viability.

For this analysis, a sensitivity analysis was conducted using RStudio to evaluate the impact of various parameters on the price PPPY. Each parameter was varied by ± 20%, and the resulting changes in outcomes were analysed to identify the most influential cost drivers.

### Parameters & operationalisation

To operationalise the pricing model and the outlined scenarios, several assumptions must be made, based on the development trajectory of repurposed bevacizumab and relevant scientific literature.

#### R&D costs

The minimum-cost scenarios assume that the only required expense for repurposing bevacizumab is regulatory registration via the EMA’s well-established use pathway, with no need for additional clinical trials. As of 2024, the total EMA fee for such an application, including the main dossier and mandatory fees for the rapporteur, co-rapporteur, and PRAC rapporteur, is €780.900 [58]. As this fee applies to the entire EU market, no adjustment is required in Scenario 2. However, in Scenario 1, which focuses on the Dutch market (representing roughly 4% of the EU population [59, 60]), the cost is proportionally scaled to reflect the smaller market size.

The maximum-cost scenarios assume a more complex development process, extending beyond standard biosimilar requirements. However, estimating development costs is challenging due to a lack of transparency in pharmaceutical R&D spending. Some studies include cost-of-capital and cost-of-failure, which are addressed separately in this framework, while others exclude them [61–68]. R&D costs also vary depending on product type and therapeutic area [61, 64]. Repurposed products are generally less costly to develop than NMEs, as they benefit from existing human safety data and often require fewer new trials. Some estimates suggest repurposing may cost as little as 10% of the investment required for entirely new drug development [69, 70].

The maximum-cost scenarios include an additional €50 million to reflect out-of-pocket R&D expenses beyond biosimilar-related development costs, such as comparability trials, regulatory filings, and investment in manufacturing infrastructure, which are already embedded in the manufacturing costs, benchmarked against the market price of off-label Avastin^®^. The €50 million figure corresponds to an estimated €10 million per clinical trial used to support EMA registration [23, 24].

#### Cost-of-failure

Drug development is characterised by high attrition rates, and these failures lead to significant costs that must be recouped through successfully marketed products. Although bevacizumab is a repurposed drug with inherently lower risks and associated cost-of-failure, the maximum scenarios still incorporate these expenses.

Gupta Strategists report that cost-of-failure accounts for approximately 40% of total R&D expenditure for an average NME and equates to around 5.71 times the out-of-pocket R&D costs [62]. Similarly, Sertkaya et al. report that cost-of-failure comprises, on average, 39.1% of total development costs, or 1.99 times out-of-pocket R&D costs [65]. Using these ratios, cost-of-failure is integrated into pricing as a proportion of assigned out-of-pocket R&D costs. For this analysis, cost-of-failure is estimated at 3.85 times the assigned out-of-pocket R&D costs in the maximum scenarios, a midpoint between the estimates from Gupta Strategists [62] and Sertkaya et al. [65].

#### Cost-of-capital

The cost-of-capital represents the expense of obtaining financing, combining the costs of debt (interest obligations) and equity (expected shareholder returns). It is expressed as a percentage of invested capital and is used as a discount factor in economic evaluations. Consistent with DiMasi et al. (2016) and Wouters et al. (2020) [61, 64], this study applies a real weighted average cost-of-capital (WACC) of 10,5% to capture the opportunity cost of funds committed during both the development (pre-marketing) and recoupment (post-launch) phase. As a real discount rate is applied, inflation is not modelled separately in the calculations.

#### Manufacturing costs

The manufacturing costs for repurposed bevacizumab are derived from the reported costs for the current off-label use of *Avastin*^®^ in nAMD, which amount to €35,53 per injection in the Netherlands, requiring 30 injections over the first five years [11]. This translates to an annual manufacturing cost of €213,18 per patient, applicable in both the minimum and maximum scenarios. This figure reflects the cost of off-label bevacizumab including preparation for intravitreal administration and is consistent with the prices of authorised bevacizumab biosimilars [54]. Importantly, these biosimilar prices already incorporate a wide range of development-related expenditures, such as the establishment of dedicated manufacturing lines, regulatory advice, and legal processes. These regulatory, legal, and product-development costs (Table 3) are therefore already embedded in the manufacturing-cost benchmark and remain separate from the additional R&D costs allocated in the maximum scenarios, which are applied specifically because bevacizumab’s ophthalmic use introduces further regulatory requirements [55].

#### SG&A costs

Empirical studies indicate that SG&A expenses typically represent a share of revenue comparable to that of cost of goods sold (COGS). Ledley et al. (2020) report median SG&A at 28.2% of revenue and COGS at 23.5%, while Chandra et al. (2024) find similar averages of ~ 24% and ~ 23%, respectively [71, 72]. According to Chandra et al., around 40% of SG&A spending is attributable to sales and marketing, with the remaining ~ 60% linked to other administrative and operational functions [72].

Drawing on this evidence base, SG&A in this analysis is expressed relative to manufacturing costs. In line with concerns about disproportionate pharmaceutical marketing expenditures [73] and following the approach of Uyl-de Groot and Löwenberg (2018) [35], sales-related SG&A is capped at 30% of manufacturing costs. Non-sales SG&A is assumed to amount to ~ 60% of manufacturing costs, consistent with Chandra et al. [72]. Together, these components yield a total SG&A allocation of ~ 90% of manufacturing costs.

#### Profit

In line with the AIM model, we apply a standard 8% profit margin in the minimum scenarios to balance investment costs and ensure a reasonable return on new drug development. This margin approximates the average return of the S&P 500 index (unadjusted for inflation) [34, 71]. By aligning the margin with stock market averages, investment in drug development remains competitive with traditional investments.

In the maximum scenarios, the profit margin is set at 25%. In a recent legal case brought by the Dutch foundation Farma ter Verantwoording (Pharmaceutical Accountability Foundation) against AbbVie over the price of Humira^®^, it was argued that 25% of total revenue should be the upper limit [74]. Any margin above this was characterised as “excess profit” or “surplus,” potentially limiting funding for other healthcare services. Accordingly, a 25% margin may be viewed as the maximum acceptable level before facing legal scrutiny.

#### Number of patients

According to the Dutch Ministry of Health, approximately 19.019 individuals in the Netherlands received bevacizumab treatment in 2022 and are therefore eligible for the registered product Lytenava^®^ [33]. This figure is used as the patient base for scenarios 1 and 3.

While several estimates exist for the total number of AMD patients in the EU [75, 76], specific data on nAMD patients eligible for bevacizumab are lacking. Extrapolating Dutch figures to the broader EU population suggests around 475.475 potential patients. However, the Netherlands administers a comparatively high number of bevacizumab injections due to its off-label inclusion in treatment guidelines, which may lead to an overestimate of the eligible EU patient pool. To reflect this, the patient population in scenarios 2 and 4 is conservatively estimated at 380.000.

## Results

**Fig. 2:**
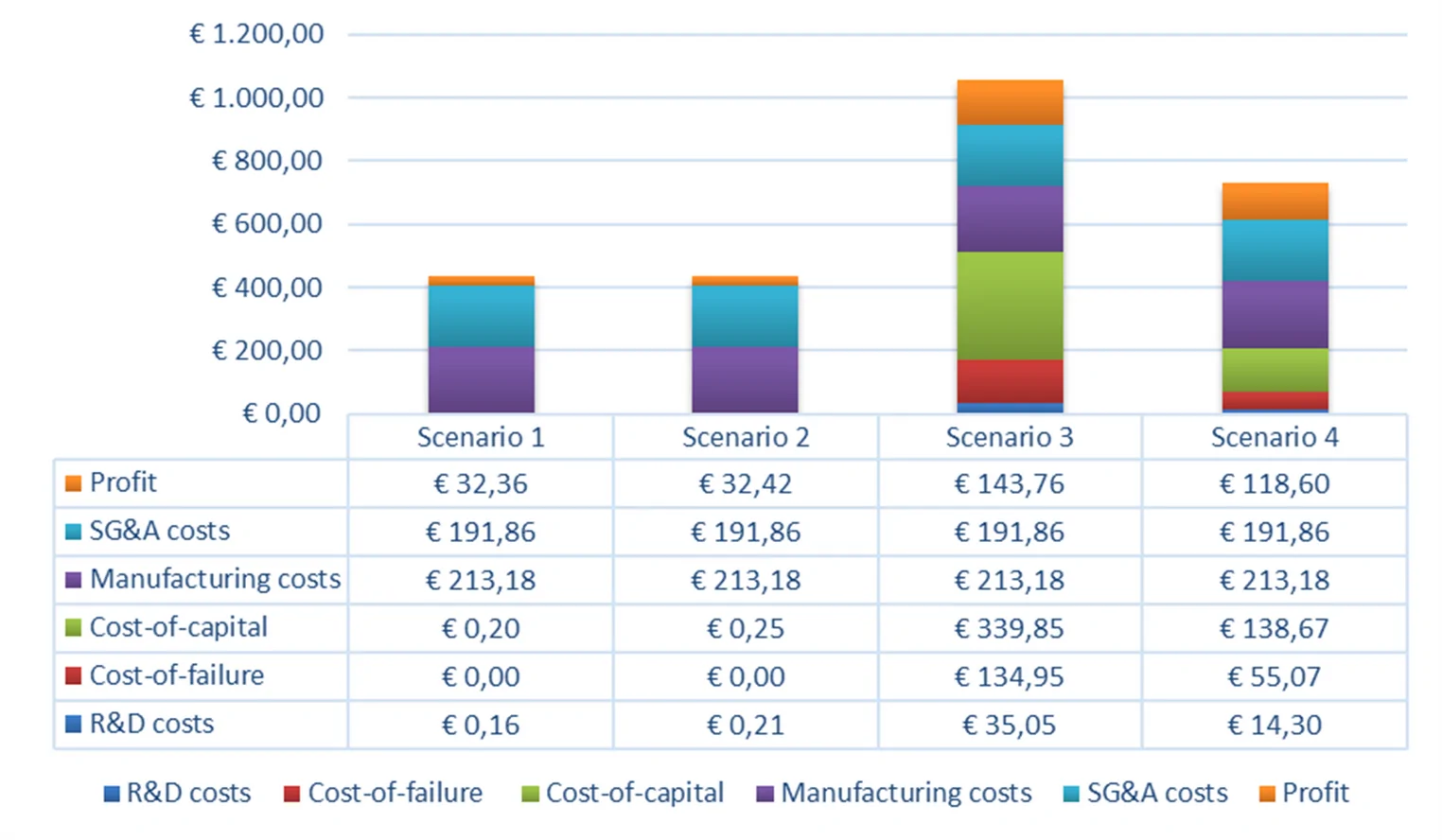
Breakdown of cost components’ relative influence final price PPPY

From the operationalisation of the pricing framework, a price PPPY ranging from €438 to €1059 is derived, translating to approximately €73 to €177 per injection, assuming six injections per year following treatment guidelines [11, 15]. This benchmark provides an estimate of a sustainable price for repurposed bevacizumab for nAMD treatment and offers insight into the key cost drivers that shape this price range (Table 5). Across all modelled scenarios, except for scenario 3, manufacturing costs represent the largest cost component, as also illustrated in Fig. 2, which shows the relative contribution of each component to the final price PPPY. Only in Scenario 3 does cost-of-capital represent the largest component. By contrast, R&D and cost-of-failure remain marginal contributors to the price PPPY across all scenarios. In terms of profitability, a pharmaceutical company operating in the European market could expect annual profits ranging from approximately €12 million (Scenario 2) to €45 million (Scenario 4) during the recoupment period when repurposing bevacizumab for ophthalmic indications such as nAMD.

**Table 5:** Overview of the models’ outputs

Items:	Scenario 1:	Scenario 2:	Scenario 3:	Scenario 4:
1. Development costs				
(Net) research & development costs	€780.900	€780.900	€ 50.000.000	€ 50.000.000
Cost-of-failure	€ 0	€ 0	€ 192.500.000	€ 192.500.000
**2. Financial correction factors**				
Cost-of-capital	€ 933.451	€ 933.451	€ 484.771.889	€ 484.771.889
**3. Market-based factor**				
R&D costs, adjusted for regional market share and recoupment period*	€ 3.124	€ 78.090	€ 666.667	€ 5.435.000
Cost-of-failure, adjusted for regional market share and recoupment period*	€ 0	€ 0	€ 2.566.667	€ 20.924.750
Cost-of-capital, adjusted for regional market share and recoupment period*	€ 3.734	€ 93.345	€ 6.463.625	€ 52.694.704
**4. Annual recurring cost items**				
Manufacturing costs	€ 4.054.470	€ 81.008.400	€ 4.054.470	€ 81.008.400
SG&A costs	€ 3.649.023	€ 72.907.560	€ 3.649.023	€ 72.907.560
**5. Profit elements**				
Profit	€ 616.529	€ 12.319.524	€ 2.734.207	€ 45.068.928
Innovation bonus	€ 0	€ 0	€ 0	€ 0
**6. Outcomes**				
Price PPPY	€ 438	€ 438	€ 1.059	€ 732
Price per injection**	€ 73	€ 73	€ 177	€ 122

* Reflects the amount that needs to be recouped annually during the recoupment period

** Assuming 6 injection/year

**Fig. 3:**
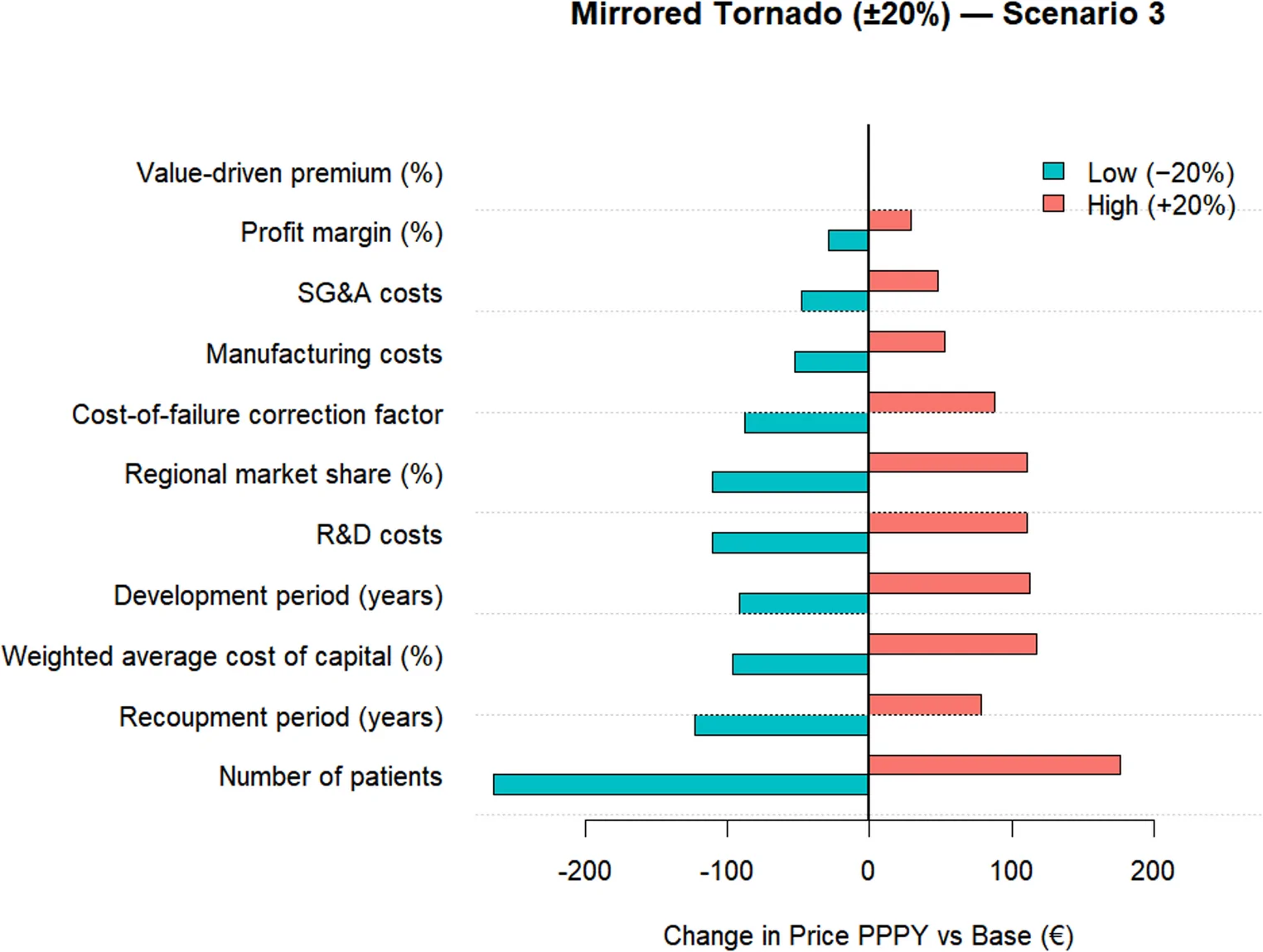
Sensitivity analyses for scenario 3

Sensitivity analyses, conducted in RStudio (Annex 1) for Scenario 3, the scenario with the highest price, illustrate both the direction and magnitude of each parameter’s impact on the price PPPY (Fig. 3). The bars represent the effects of a 20% increase or decrease in each parameter. The chart shows that the number of patients is the most sensitive parameter, followed by the recoupment period. This highlights the pivotal role of patient population size in shaping the price PPPY. A smaller patient population increases the price by dividing fixed development costs across fewer individuals, thereby raising the per-patient burden; conversely, a larger patient base lowers the price by spreading these fixed costs across more individuals. In addition, the number of patients directly affects total manufacturing costs: fewer patients reduce overall production needs, while more patients increase manufacturing volumes. As SG&A costs in this model are calculated as a percentage of manufacturing costs, changes in patient numbers therefore also cascade into SG&A expenditures. Consequently, patient population size influences not only the allocation of fixed development costs, but also variable cost components, reinforcing its central importance within the cost-based (plus) pricing framework.

Meanwhile, factors related to fixed development costs (such as the assigned R&D costs, the cost-of-failure correction factor, and the WACC), as well as market-based parameters (which scale these costs to the regional market share and recoupment period), exert a more moderate influence on the price PPPY. Importantly, variations in the profit margin have only a limited effect within the tested ranges. Taken together, these results show that the number of patients represents the principal lever determining the feasible and sustainable pricing of repurposed bevacizumab.

## Discussion

This study demonstrates how a cost-based-plus pricing approach can be applied to a repurposed bevacizumab formulation, offering valuable insights into sustainable pricing strategies for repurposed products within the pharmaceutical market. It is important to clarify that the aim of this research is not to define an exact price for repurposed bevacizumab or *Lytenava*^®^, but rather to illustrate how a transparent and structured framework can support the estimation and discussion of a socially acceptable price for such therapies. At the same time, the findings clearly show that the projected price proposed by Outlook Therapeutics cannot be justified and places an unnecessary strain on already limited healthcare budgets.

Naturally, questions may arise concerning the assumptions underpinning this model. A key limitation is the lack of cost transparency, which necessitates the use of broad estimates. However, bevacizumab is a particularly suitable case for applying cost-based-plus pricing because several major sources of uncertainty are minimized. The intravitreal use of off-label bevacizumab is well established in routine clinical practice, and reliable data exist on patient numbers currently receiving this treatment. In addition, the manufacturing costs in this model are benchmarked against the prices of existing bevacizumab biosimilars, which already incorporate costs of production setup, regulatory documentation, and quality and legal infrastructure. As a result, many of the development related cost components that can introduce uncertainty in pricing analyses are already accounted for. The remaining cost considerations therefore primarily concern the incremental evidence required to obtain regulatory approval for use in nAMD.

At the same time, there all still elements that require cautious interpretation. In particular, the maximum cost scenarios presented here are likely to overstate the actual costs associated with repurposing bevacizumab for nAMD. Given bevacizumab’s long-standing off-label use for intravitreal injections, it is reasonable to assume that fewer (pre)clinical studies were required, and that associated development risks were significantly lower than for a novel product. As such, the development costs have likely been overestimated. In the maximum scenarios, nearly €750 million in total development costs, including cost-of-capital, are assigned. This amount most likely exceeds the costs of developing a new formulation of a well-known monoclonal antibody. Even then, the resulting cost-based plus price remains significantly lower than the projected value-based price of *Lytenava*^®^, illustrating that the price Outlook Therapeutics is asking for their product places an undue strain on healthcare systems. Furthermore, there is room for debate on whether costs-of-failure should be included at all for a product with established off-label use, and to what extent health payers should bear the cost of inefficiencies in drug development through cost-based pricing. On the other hand, the fact that *Lytenava*^®^ failed to meet the prespecified noninferiority endpoint at week 8 in the NORSE EIGHT trial [77, 78], despite being a well-established compound, highlights the real risk of late-stage failure when developing biological products.

There are also broader considerations regarding the R&D costs assigned in the maximum scenarios, which are based on the premise that this involves a biosimilar intended for ophthalmology rather than oncology. One could argue that the established use of *Avastin*^®^ in ophthalmology could serve as a reference for registering any bevacizumab product for nAMD under the well-established use pathway. This raises questions about the necessity of conducting extensive additional clinical trials and assigning the associated costs as assumed in the maximum scenarios. Moreover, using the current price of off-label bevacizumab as a proxy for manufacturing costs likely overestimates true production costs, as this price not only implicitly reflects development-related cost items included in the biosimilar market price, but also already includes preparation for intravitreal use and a producer’s profit margin. Similarly, the assumed 10-year development period in the maximum scenarios likely overstates the timeline for developing a new repurposed formulation of bevacizumab. Repurposed products typically have shorter development cycles due to existing safety and efficacy data. Additionally, SG&A costs may also have been overestimated, as bevacizumab is already included in clinical guidelines and is widely familiar to ophthalmologists, reducing the need for aggressive marketing and consequently the cost-of-sales. All of this suggests that the total assigned costs are likely an overestimation, implying that the final price PPPY for a repurposed formulation of bevacizumab could be even lower than projected.

Beyond the assumptions about development costs, there are also broader uncertainties regarding patient numbers, which further nuance the estimated price PPPY. The model does not adjust the projected patient population for mortality or incidence trends. Studies on the prevalence of AMD in Europe suggest that the number of individuals affected by late-stage AMD, including nAMD, will rise significantly due to population ageing [75, 76]. As a result, the pool of eligible patients is expected to grow, potentially reducing the cost per patient over time. On the other hand, predicting the market share a registered repurposed bevacizumab might capture remains uncertain, as it depends on factors such as the competitive landscape, pricing strategies, reimbursement policies, and both prescriber and patient preferences. However, the success of off-label bevacizumab use in the Netherlands clearly demonstrates a strong demand for cost-effective treatments for nAMD. This suggests that a registered and affordable version of bevacizumab could potentially capture a significant share of the market, especially given the pressure to reduce healthcare costs and the positive experiences of both clinicians and patients with the drug.

Finally, the scenarios presented here assume a recoupment period that is shorter than the full patent-protected period. If no competitive products (e.g. more effective alternatives) enter the market during this time, the initial investment could be recouped before the end of the pricing period designed to recover those costs. This suggests an opportunity to negotiate agreements that limit the duration for which the higher price applies, aligning pricing more closely with the recoupment period and ensuring fairer cost recovery.

Regarding these uncertainties, it is important to reflect on the results from the sensitivity analysis for Scenario 3 and the breakdown of each cost component’s relative influence on the final price PPPY. Manufacturing costs and the number of patients clearly stand out as influential and sensitive parameters. And although these parameters may also be subject to over- or underestimation, as mentioned above, they can be determined with relatively greater certainty given that bevacizumab has been prescribed to patients for many years at well-established costs.

This model not only demonstrates that the projected price is unnecessarily high, but also offer the advantage of providing deeper insight into price structure and underlying assumptions. By explicitly breaking down the cost components, the model allows for critical evaluation of each element, enabling health payers to challenge inflated or unjustified prices. For instance, if Outlook Therapeutics Ltd. were to request a higher price for *Lytenava*^®^, this type of cost breakdown would allow payers to assess whether the price is justifiable. To justify a higher price, the company would then need to clearly explain any differing assumptions that lead to the proposed pricing. Greater transparency in disclosing actual costs and their relevance could accelerate payer acceptance and reimbursement decisions. The model thus supports a more informed and collaborative dialogue between health payers and pharmaceutical developers, particularly in the context of drug repurposing. However, it should be noted that this would be unnecessary if mandatory transparency of real costs incurred were the basis of registration for such non-inventive products, which are essentially built upon the ideas of others.

As R&D costs are often not publicly disclosed [79], accurate assessments are difficult. Furthermore, there is frequent ambiguity about which costs are included under R&D. This lack of transparency limits the ability of healthcare systems and payers to understand the rationale behind pricing decisions, thereby weakening their negotiating position. The proposed framework helps to open this discussion by promoting transparency and providing a structure for evaluating cost breakdowns. By critically assessing each component, both parties can work towards a price that is socially acceptable, while still supporting sustainable profit margins essential for incentivising further drug development. This incentive is not only necessary to recoup investments made to register known uses, but also to encourage the exploration of new indications for older, low-cost drugs.

Despite the promise of delivering new treatment options, drug repurposing remains underexploited. Manufacturers pursuing new indications often face disincentives, such as low prices, generic competition, or cross-label referencing by other brands [80, 81], making investment in repurposing financially unattractive. On the other hand, when successful, repurposing can create monopolies that allow companies to set high prices, which may place an undue burden on healthcare systems. It is therefore essential to develop appropriate incentives that encourage investment in repurposing while maintaining affordability. A purely publicly funded route for all repurposing efforts is unrealistic, given the vast number of potentially eligible drugs. Incentives should also support clinical research into new indications, not just the replication of known uses for regulatory purposes—especially when such studies may not be strictly necessary.

One recently proposed policy solution is to legislate exclusive reimbursement for repurposed indications [82]. Under this model, only the specifically repurposed brand would be reimbursed for the new indication, excluding alternative brands and thus creating a more predictable business case for developers. To maintain affordability, this policy would need to be accompanied by pricing policies that ensures fairness and transparency for all parties involved. This article demonstrates how pricing for repurposed products can be approached through a transparent, cost-based model.

## Supplementary Information

Below is the link to the electronic supplementary material.

Supplementary Material 1 (DOCX 31.9 KB)

## Data Availability

All data analysed during this study are included in this published article and its annex.
