# Supplementary material for: Repurposing biosimilars, rethinking costs: a framework for sustainable drug pricing for repurposed bevacizumab for intravitreal injections
Source: Eur J Health Econ. 2026 Feb 7;27(7):1453–67. doi: 10.1007/s10198-025-01891-3 (PMC13619761; doi:10.1007/s10198-025-01891-3)
Supplement: Supplementary file 1 [file 10198_2025_1891_MOESM1_ESM.docx]

## Annex 1: R-scripts for sensitivity analysis:

############################################################

```
## Price PPPY (Eq1–Eq11) + ±20% One-Way Sensitivity       ##
## Scenario 3 only | Abs. MC & SG&A | Breakdown & CSVs    ##
```

############################################################

\## -------- User options --------

SENSITIVITY_PCT <- 0.20	# ±20%

options(stringsAsFactors = FALSE, scipen = 999)

\## -------- Helpers --------

`%na0%` <- function(x, y = 0) ifelse(is.na(x), y, x)

as_pct <- function(x) { x <- suppressWarnings(as.numeric(x)); x <- ifelse(is.na(x),0,x); ifelse(x>1,x/100,x) }

ensure_dir <- function(path) { d <- dirname(path); if (!dir.exists(d)) dir.create(d, recursive = TRUE, showWarnings = FALSE) }

euro_fmt <- function(x) formatC(x, format="f", digits=0, big.mark=",")

\## -------- Core calculator (returns detailed pieces for overview & breakdown) --------

calc_price_pppy <- function(df_inputs) {

with(df_inputs, {

\# CoF from factor (or absolute if supplied)

cof_from_factor <- as.numeric(`Cost-of-failure correction factor` %na0% 0) * as.numeric(`R&Dᵢ` %na0% 0)

`CoFᵢ_eff` <- if ("CoFᵢ" %in% names(df_inputs) && !all(is.na(df_inputs$`CoFᵢ`))) {

as.numeric(df_inputs$`CoFᵢ` %na0% 0)

} else { cof_from_factor }

\# Cost of capital (development + recoupment) — inflation handled via GDPᵢ in both phases

wacc <- as_pct(as.numeric(`WACCᵢ` %na0% 0))

gdp <- as_pct(as.numeric(`GDPᵢ` %na0% 0))

D	<- as.numeric(`Dᵢ` %na0% 0)

R	<- as.numeric(`Rᵢ` %na0% 0)

base_RD_CoF <- as.numeric(`R&Dᵢ` %na0% 0) + `CoFᵢ_eff`

CoC_Development_vec <- base_RD_CoF * ((1 + wacc)^D * (1 + gdp)^D - 1)

CoC_Recoup_vec <- vapply(seq_along(R), function(i) {

if (R[i] <= 0) return(0)

sum((base_RD_CoF[i] + CoC_Development_vec[i]) *

(1 - (1:R[i]) / R[i]) * wacc[i] * (1 + gdp[i])^(1:R[i]))

}, numeric(1))

CoC_vec <- CoC_Development_vec + CoC_Recoup_vec

\# Market allocation & annual cost items

MAF_vec <- as_pct(as.numeric(`Regional market share` %na0% 0)) / pmax(R, 1)

if (!"Manufacturing costs" %in% names(df_inputs)) stop("Need 'Manufacturing costs' column (absolute €).")

MC_vec	<- as.numeric(df_inputs$`Manufacturing costs` %na0% 0)

SGA_vec <- as.numeric(df_inputs$`SG&Aᵢ` %na0% 0)

\# Basis for profit & innovation (Eq10–11)

basis_vec <- ((as.numeric(`R&Dᵢ`) + `CoFᵢ_eff`) * MAF_vec) + MC_vec + SGA_vec

profit_m <- as_pct(as.numeric(`Profit margin` %na0% 0))

IB_m	<- as_pct(as.numeric(`Value-driven premium` %na0% 0))

P_vec	<- profit_m * basis_vec

IB_vec	<- IB_m	* basis_vec

\# Numerator for Eq1 and PPPY

numer_vec <- ((as.numeric(`R&Dᵢ`) + `CoFᵢ_eff` + CoC_vec) * MAF_vec) +

MC_vec + SGA_vec + P_vec + IB_vec

denom	<- pmax(as.numeric(`Patientsᵢ` %na0% 0), 1)

PPPY	<- sum(numer_vec) / sum(denom)

list(

Price_PPPY = PPPY,

RnD	= sum(as.numeric(`R&Dᵢ` %na0% 0)),

CoF	= sum(`CoFᵢ_eff`),

RnD_CoF	= sum(as.numeric(`R&Dᵢ` %na0% 0) + `CoFᵢ_eff`),

CoC_dev	= sum(CoC_Development_vec),

CoC_rec	= sum(CoC_Recoup_vec),

CoC_total	= sum(CoC_vec),

MAF	= mean(MAF_vec),

RnD_MAF	= sum(as.numeric(`R&Dᵢ` %na0% 0) * MAF_vec),

CoF_MAF	= sum(`CoFᵢ_eff` * MAF_vec),

CoC_MAF	= sum(CoC_vec * MAF_vec),

MC	= sum(MC_vec),

SGA	= sum(SGA_vec),

Basis_sum	= sum(basis_vec),

Profit_amt = sum(P_vec),

Innov_amt	= sum(IB_vec),

CoF_sum	= sum(`CoFᵢ_eff`),

CoC_sum	= sum(CoC_vec)

)

})

}

\## -------- One-way sensitivity --------

one_way_sa <- function(df_inputs, vary_fields, pct = 0.20) {

base <- calc_price_pppy(df_inputs)$Price_PPPY

res <- lapply(vary_fields, function(fld) {

low_df <- df_inputs; high_df <- df_inputs

scale_field <- function(x, mult) { if (is.numeric(x)) return(x*mult); nx <- suppressWarnings(as.numeric(x)); if (all(!is.na(nx))) return(nx*mult); x }

low_df[[fld]] <- scale_field(low_df[[fld]], 1 - pct)

high_df[[fld]] <- scale_field(high_df[[fld]], 1 + pct)

low <- calc_price_pppy(low_df)$Price_PPPY

high <- calc_price_pppy(high_df)$Price_PPPY

data.frame(Component=fld, Base=base, Low=low, High=high, Low_Δ=low-base, High_Δ=high-base, check.names=FALSE)

})

do.call(rbind, res)

}

\## -------- Labels (for plots) --------

label_map <- c(

"R&Dᵢ" = "R&D costs",

"Cost-of-failure correction factor" = "Cost-of-failure correction factor",

"WACCᵢ" = "Weighted average cost of capital (%)",

"Dᵢ" = "Development period (years)",

"Regional market share" = "Regional market share (%)",

"Rᵢ" = "Recoupment period (years)",

"Manufacturing costs" = "Manufacturing costs",

"SG&Aᵢ" = "SG&A costs",

"Profit margin" = "Profit margin (%)",

"Value-driven premium" = "Value-driven premium (%)",

"Patientsᵢ" = "Number of patients"

)

apply_labels <- function(sa) { sa$Display <- label_map[sa$Component]; sa$Display[is.na(sa$Display)] <- sa$Component; sa }

\## -------- Plotters --------

save_abs_tornado <- function(sa, pct, scenario_tag, out_dir = ".") {

sa <- apply_labels(sa); effect <- pmax(abs(sa$Low_Δ), abs(sa$High_Δ))

ord <- order(effect, decreasing = TRUE); sa <- sa[ord, ]; effect <- effect[ord]

png_file <- file.path(out_dir, paste0("scenario_", scenario_tag, "_tornado_abs.png")); ensure_dir(png_file)

png(png_file, width=1200, height=800, res=120); op <- par(mar=c(7,22,6,4))

barplot(effect, horiz=TRUE, names.arg=sa$Display, las=1,

xlab="Absolute change in Price PPPY (€)",

main=paste0("Tornado (absolute, ±", round(100*pct), "%) — Scenario ", toupper(scenario_tag)))

grid(nx=NA, ny=NULL); par(op); dev.off(); png_file

}

save_mirrored_tornado <- function(sa, pct, scenario_tag, out_dir = ".") {

sa <- apply_labels(sa); effect <- pmax(abs(sa$Low_Δ), abs(sa$High_Δ))

ord <- order(effect, decreasing = TRUE); sa <- sa[ord, ]

left_vals <- -abs(sa$Low_Δ); right_vals <- abs(sa$High_Δ); vals <- rbind(left_vals, right_vals)

x_max <- max(abs(vals), na.rm=TRUE)*1.05

png_file <- file.path(out_dir, paste0("scenario_", scenario_tag, "_tornado_mirrored.png")); ensure_dir(png_file)

png(png_file, width=1400, height=900, res=120); op <- par(mar=c(9,28,7,6))

bp <- barplot(vals, horiz=TRUE, beside=TRUE, col=c("#00BFC4","#F8766D"),

xlim=c(-x_max,x_max), names.arg=rep("", length(left_vals)*2), las=1,

xlab="Change in Price PPPY vs Base (€)",

main=paste0("Mirrored Tornado (±", round(100*pct), "%) — Scenario ", toupper(scenario_tag)))

grid(nx=NA, ny=NULL); abline(v=0,lwd=2); y_centers <- colMeans(bp)

axis(2, at=y_centers, labels=sa$Display, las=1, tick=FALSE)

legend("topright", inset=0.02, legend=c(paste0("Low (−",round(100*pct),"%)"), paste0("High (+",round(100*pct),"%)")),

fill=c("#00BFC4","#F8766D"), bty="n")

par(op); dev.off(); png_file

}

\## -------- Component breakdown (table + plot) --------

build_breakdown <- function(df) {

c <- calc_price_pppy(df)

data.frame(

Item = c(

"R&D costs",

"Cost-of-failure",

"R&D costs plus cost-of-failure",

"Cost-of-capital (development phase), adjusted for inflation",

"Cost-of-capital (recoupment phase), adjusted for inflation",

"Cost-of-capital (development & recoupment phase), adjusted for inflation",

"R&D costs, adjusted for market size and recoupment period*",

"Cost-of-failure, adjusted for market size and recoupment period*",

"Cost-of-capital (development & recoupment phase), adjusted for market size, recoupment period, and inflation*",

"Manufacturing costs",

"Sales, general, and administrative (SG&A) costs",

"Profit",

"Innovation bonus",

"Price PPPY"

),

EUR = c(

c$RnD, c$CoF, c$RnD_CoF, c$CoC_dev, c$CoC_rec, c$CoC_total,

c$RnD_MAF, c$CoF_MAF, c$CoC_MAF, c$MC, c$SGA, c$Profit_amt, c$Innov_amt, c$Price_PPPY

),

check.names = FALSE

)

}

save_breakdown_plot <- function(df, scenario_tag, out_dir = ".") {

tb <- build_breakdown(df)

png_file <- file.path(out_dir, paste0("scenario_", scenario_tag, "_component_breakdown.png"))

ensure_dir(png_file)

ord <- seq_len(nrow(tb))

vals <- tb$EUR[ord]; names(vals) <- tb$Item[ord]

xmax <- max(vals[1:13], na.rm = TRUE) * 1.15 # scale by totals, not PPPY

png(png_file, width=1200, height=900, res=120)

op <- par(mar=c(8, 32, 6, 4))

bp <- barplot(vals, horiz=TRUE, las=1,

names.arg = names(vals),

xlim = c(0, xmax),

xlab = "Euros (€)",

main = paste0("Component Breakdown — Scenario ", toupper(scenario_tag)))

grid(nx=NA, ny=NULL)

text(x = vals + xmax*0.01, y = bp, labels = euro_fmt(vals), adj = 0, cex = 0.8)

mtext("* Adjusted by regional market share and recoupment period (MAF).", side=1, line=5, cex=0.9)

par(op); dev.off()

utils::write.csv(tb, file = paste0("scenario_", scenario_tag, "_component_breakdown.csv"), row.names = FALSE)

png_file

}

```
## -------- Build Scenario 3 from the image --------
## From the table:
## - R&D = €50 million
## - Cost-of-failure = 3.85 × R&D
## - WACC = 10.5%
## - Development period = 10 years
## - Regional market share = 4%
## - Recoupment period = 3 years
## - Manufacturing costs: €35.53 per tablet × 6 injections pppy  =>  €213.18 per patient per year
## - SG&A = 90% of MC (absolute)
## - Profit margin = 25%
## - Innovation bonus = 0%
## - Patients = 19,019
```

build_scn3 <- function() {

patients <- 19019

mc_per_patient <- 35.53 * 6	# €213.18 pppy

MC <- mc_per_patient * patients

SGA <- 0.90 * MC

data.frame(

check.names = FALSE,

`R&Dᵢ` = 50e6,

`Cost-of-failure correction factor` = 3.85,

`WACCᵢ` = 10.5,

`Dᵢ` = 10,

`Rᵢ` = 3,

`GDPᵢ` = 0,

`Regional market share` = 4,

`Manufacturing costs` = MC,

`SG&Aᵢ` = SGA,

`Profit margin` = 25,

`Value-driven premium` = 0,

`Patientsᵢ` = patients

)

}

\## -------- Sensitivity fields --------

vary_these <- c("R&Dᵢ","Cost-of-failure correction factor","WACCᵢ","Dᵢ",

"Regional market share","Rᵢ","Manufacturing costs","SG&Aᵢ",

"Profit margin","Value-driven premium","Patientsᵢ")

\## -------- Run Scenario 3, save plots/CSVs --------

scenario_tag <- "3"

df3 <- build_scn3()

\# Baseline

calc3 <- calc_price_pppy(df3)

\# Sensitivity

sa3 <- one_way_sa(df3, vary_these, SENSITIVITY_PCT)

utils::write.csv(sa3, file=paste0("scenario_", scenario_tag, "_sensitivity.csv"), row.names=FALSE)

\# Plots

save_abs_tornado(sa3, SENSITIVITY_PCT, scenario_tag)

save_mirrored_tornado(sa3, SENSITIVITY_PCT, scenario_tag)

save_breakdown_plot(df3, scenario_tag)

\# Outcome line + mini overview CSV

overview3 <- data.frame(

Scenario	= scenario_tag,

Patients	= df3$`Patientsᵢ`[1],

RnD_EUR	= df3$`R&Dᵢ`[1],

CoF_factor	= df3$`Cost-of-failure correction factor`[1],

WACC	= df3$`WACCᵢ`[1],

DevYrs	= df3$`Dᵢ`[1],

RecoupYrs	= df3$`Rᵢ`[1],

MarketShare	= df3$`Regional market share`[1],

MC_EUR	= df3$`Manufacturing costs`[1],

SGA_EUR	= df3$`SG&Aᵢ`[1],

ProfitMargin = df3$`Profit margin`[1],

ValuePremium = df3$`Value-driven premium`[1],

CoF_EUR	= calc3$CoF_sum,

CoC_EUR	= calc3$CoC_sum,

Basis_EUR	= calc3$Basis_sum,

Profit_EUR	= calc3$Profit_amt,

Innovation_EUR= calc3$Innov_amt,

Price_PPPY	= calc3$Price_PPPY

)

utils::write.csv(overview3, file="outcomes_overview_scenario_3.csv", row.names=FALSE)

cat("Scenario 3 — Price PPPY: ", formatC(calc3$Price_PPPY, format="f", digits=2, big.mark=","), " €\n", sep="")

cat("Saved: scenario_3_sensitivity.csv, scenario_3_tornado_abs.png, scenario_3_tornado_mirrored.png\n")

cat("Saved: scenario_3_component_breakdown.png and scenario_3_component_breakdown.csv\n")

cat("Saved: outcomes_overview_scenario_3.csv\n")
